# Nurses’ Experiences of Caring for Patients With Suspected or Confirmed COVID-19 in the Initial Stage of the Pandemic

**DOI:** 10.1177/23779608221114981

**Published:** 2022-07-21

**Authors:** Janet Mattsson, Elin Hedlund, Lisa George-Svahn, Elina Scheers-Andersson, Monir Mazaheri, Gunilla Björling

**Affiliations:** 1 Swedish Red Cross University, Stockholm, Sweden; 2 Department of Learning, Informatics, Management and Education, Karolinska Institutet, Stockholm, Sweden; 3 101099 Rinkeby Primary Care Center, Stockholm, Sweden; 4 Department of Neurobiology, Care Sciences and Society, 411412 Karolinska Institutet, Stockholm, Sweden; 5 108094 Kilimanjaro Christian Medical University College, Moshi, Tanzania

**Keywords:** COVID-19, qualitative research, experiences, primary care, practice, advance practice nurses

## Abstract

**Introduction:**

Nursing staff have faced various challenges during the global pandemic of COVID-19 such as nursing shortages. The great number of COVID-19 patients requiring hospitalization placed heavy demands on healthcare staff to maintain patient safety and to work according to constantly changing guidelines to prevent the spread of infection.

**Objective:**

The objective was to describe nurses’ experiences of caring for patients with suspected or confirmed COVID-19 in the initial phase of the pandemic.

**Methods:**

The study has a qualitative design. Semi-structured interviews were conducted with seven nurses in primary care and hospital care during the initial stage of the pandemic. Qualitative content analysis with an inductive approach was used.

**Results:**

The nurses expressed that the working routines changed very quickly at the onset of the pandemic. A triage system was implemented to care for patients with symptoms of COVID-19 to prevent transmission between patients. A major change was the constant use of personal protective equipment in patient care. The nurses also experienced a sense of inadequacy regarding the care of the patients and became emotionally affected and exhausted.

**Conclusion:**

The nurses experienced that many patients worsened clinically, leading to exhausting and difficult nursing care situations. They also experienced increasing responsibility since new protective equipment and procedures needed to be quickly implemented according to frequently changing recommendations, causing the nurses to feel uncertain about how to maintain patient safety. Support from colleagues was crucial to cope throughout the initial stage of the pandemic.

## Background

Nursing staff have faced various challenges during the global pandemic of COVID-19 along with previous challenges such as nursing shortages. Nurses have in general described a severe lack of resources including personal protective equipment, staffing, and testing as the main challenges during the COVID-19 pandemic (Halcomb et al., 2020; White et al., 2021).

The pandemic affected nurses and their work conditions differently depending on their care setting. The primary healthcare nurses experienced job insecurity and the threat of losing employment altogether with an overall decrease in their working hours (Halcomb et al., 2020). They also experienced a negative impact on their safety since adequate protective equipment was not available. Furthermore, nurses could not provide the same quality of care compared to pre-pandemic conditions. Nurses in nursing homes experienced not only physical and mental restrains and burnout, but also blame and negative image of their efforts in the media which reported on the sub-optimal conditions in nursing homes (White et al., 2021) since nursing homes were one of the hardest-hit settings by the COVID-19. Nursing homes and their staff were often described as poorly equipped to manage the virus and staff were portrayed as incompetent. Moreover, with intensified nursing staff shortages and rapidly changing guidelines and working conditions, nurses themselves experienced a wide range of symptoms while working under pandemic conditions.

### Review of Literature

Italian nurses who cared for patients with COVID-19 experienced psychological pressure and high levels of emotional exhaustion (Barello et al., 2020). A study in France showed that staff reported symptoms of anxiety, depression, and post-traumatic stress disorder (PTSD) and needed sleeping pills or anti-anxiety medications (Flateau et al., 2021). Canadian nurses relayed the following factors as contributors to their psychological distress: trying to keep themselves safe while taking care of critically ill patients, struggling to take care of personal commitments to themselves and their families, constantly changing policy and information as well as unclear and frequent messaging (Crowe et al., 2021). These nurses provided direct care to patients in critical care units, and reported poor mental health specific to symptoms of PTSD, depression, and psychological distress (Crowe et al., 2021). Among other symptoms that nurses experienced as a consequence of working during the pandemic were frequent somatic symptoms including increased irritability, sleep disturbances, and muscle tension (Barello et al., 2020). Some gender differences have been found in the reported experiences of healthcare staff. Regenold and Vindrola-Padros (2021) write that female caregivers had tougher experiences during the first pandemic peak in England in 2020.

In Sweden, the number of infected people was high at the beginning of the pandemic compared to other European countries, especially compared with the other Nordic countries. Unlike many other countries, Sweden did not go into lockdown and kept many services open such as schools, which resulted in a high incidence of COVID-19. According to data on the COVID-19 situation published on May 10, 2020, by the Public Health Agency in Sweden (2020a), the incidence of COVID-19 was 26,322, whereof 3,225 persons were diseased, thus the death rate in May 2020 was over 12.25% (Public Health Agency, 2020b). However, due to the high number of infected people, society could not test all of them, that is, people with mild symptoms so testing was restricted to patients in hospital settings. The number of infected persons was therefore probably much higher. Furthermore, on May 10, 2020, there were 1,753 persons who were cared for in intensive care units in the country. The recommendations from the public health authorities were to use basic infection disease protective precautions, isolate infected people, and identify those with severe symptoms in need of hospital care (Public Health Agency Sweden, 2020c). Moreover, there was no general recommendation to wear a face mask in public spaces. Sweden has around 10 million inhabitants.

Our literature review shows that even though there is ample literature describing the experience of healthcare professionals including nurses in providing care during the COVID-19 pandemic, qualitative exploratory studies on the Swedish population are scarce. In particular, we do not know much about the experience of nurses in the very first weeks of the outbreak in Sweden. The current study describes the first encounters of Swedish nurses in primary- and hospital care with patients with suspected or confirmed COVID-19 and reports their experiences of providing care to this patient group. To our knowledge, there is no other similar study in a Swedish context. Therefore, the aim of this study was to describe nurses’ experiences of taking care of patients with suspected or confirmed COVID-19 in the initial phase of the pandemic.

## Methods

### Study Design

This study emerged from an empirical context with an inductive approach and qualitative design. According to Polit and Beck (2017), a qualitative method means that a phenomenon is sought after with a focus on understanding life experience. The method was chosen as the most suitable method since this study focuses on exploring registered nurses’ (RN) experience of caring for patients with suspected or confirmed COVID-19 infection. The study follows the consolidated criteria for reporting qualitative research, COREQ (Tong et al., 2007). The study report follows the SON-Original-Research-Guideline.

### Research Question

What is the experience of nurses who were taking care of patients with suspected or confirmed COVID-19 at the beginning of the pandemic 2020?

### Sample

This qualitative interview study was performed between March and April 2020 in Stockholm, Sweden. Interviews were conducted at three different sites at the onset of the COVID-19 epidemic. A convenience sample was used to approach registered nurses who could provide us with firsthand experiences of caring for patients with suspected or confirmed COVID-19 infection. Convenience sampling entails choosing informants based on their availability and willingness to participate in the interviews (Polit & Beck, 2017).

Registered nurses working in three different healthcare settings within an urban region in Sweden; two primary care centers whereof one in a high socio-economic area and the other in an area with low socioeconomic status, and one inpatient COVID-19 ward (former internal medicine ward) at a large regional hospital), were asked to participate in the interview study. In total 12 RNs were asked to participate in the study; seven of them accepted, two RNs declined, and three RNs did not respond. All informants provided informed consent and were informed that their participation was voluntary and could end participation at any time without reason.

### Inclusion Criteria

Inclusion criteria for participating in this study were registered nurses, working with patients with suspected or confirmed COVID-19 infection, with at least 6 months of prior work experience either from inpatient- or outpatient nursing care.

### Exclusion Criteria

Exclusion criteria were registered nurses who did not work with patients suspected or positive for COVID-19 and/or with less than 6 months of work experience.

### Data Collection

Semi-structured interviews with open thematic questions were selected as the data collection method since interviews can generate rich narratives from informants (Polit & Beck, 2017). An interview guide was used and consisted of the following themes: *working with patients with COVID-19 infection, guidelines for the use of protective equipment, feelings about the current situation*, and *priorities*.

During the interview, additional probing questions were asked to explore the informant's experiences of caring for patients with COVID-19. The chosen interview technique means that the interviewer aims to obtain as much information as possible by allowing informants to explain and express themselves openly (Polit & Beck, 2017). Interviews were conducted on the online platform Zoom due to pandemic restrictions. A pilot interview was recorded and lasted 65 min. Both the interviewer and the informants had their video cameras on to be able to see each other and communicate as normally as possible. Furthermore, using a camera allowed the interviewer to follow the informant during the interview and observe how the informant reacted to various questions. There was one occasion where the camera did not work but since the sound worked well the interview could still be held. All informants sat in a quiet environment at home or at work during the interview. The interviews were conducted with one informant at a time and lasted between 20 and 70 min until saturation occurred. The interviews were recorded via a function in Zoom and on a mobile phone to facilitate the verbatim transcription of the interviews afterward. All interviews were transcribed verbatim immediately after each interview.

### Data Analysis

Transcripts were compared to the recordings and read several times to ensure accuracy and that they captured the informant's experiences expressed in the interview situation. The informants were also given the opportunity to read through the transcription of their own interviews for validation or clarification (Polit & Beck, 2017).

The texts were then analyzed through qualitative content analysis with an inductive approach (Graneheim & Lundman, 2004). The coding process was first performed by the second author and then evaluated by the entire research group. The *first* step entailed reading and rereading to grasp a sense of the whole, that is, what was in the foreground and what was in the background. In the *second* step, meaning units were created, and the parts that contained information relevant to the study were marked, condensed, given a code, and then placed into subcategories. In the *third* step, the subcategories were re-analyzed and compiled into categories, with each category expressing something specific about the content. In the *fourth* and last step of the analysis, meaningful units, subcategories, and categories were analyzed to find the latent content and finally based on these findings, themes emerged. The analysis process is not a straightforward process but rather an iterative process that revisits the text, categories, and themes several times to ensure the conformability of findings (Graneheim & Lundman, 2004).

### Institutional Review Board Approval—Ethical Considerations

The study was conducted according to the Swedish Ethical Board- and the Swedish Red Cross Uniiversity's ethical guidelines, as well as to the Declaration of Helsinki to protect the participants’ rights in the study (World Medical Association, 2018). Permission to conduct the study was requested and obtained by the university ethical board and the heads of the three clinics involved in the study. All informants were 18 years of age or older. Informed written consent was obtained from all informants. Participation was voluntary and the participants could cease their participation at any time. Informants were given the opportunity to receive interview questions prior to the interview, as well as comment on the transcribed interviews afterward. Furthermore, informants were informed that all data collection would be handled confidentially and stored for 10 years on a USB in a locked safe.

## Results

### Sample Characteristics

Four female and three male registered nurses participated in the present study. The number of years working as a nurse ranged from 1.5 to 29 years. Descriptive information about the study sample is presented in Table 1.

**Table 1.** Descriptive Information About the Participants.

Informant	Gender	Years as RN	Location of work
Nurse 1	Male	21	COVID ward
Nurse 2	Male	3	Primary care center/homecare
Nurse 3	Female	11	Primary care center
Nurse 4	Male	1.5	COVID ward
Nurse 5	Female	12	Primary care center/homecare
Nurse 6	Female	29	Primary care center
Nurse 7	Female	8	COVID ward

### Research Question Results

The results of the study describe how registered nurses from different healthcare settings experienced the care of patients with COVID-19 at the onset of the pandemic. The themes of *Challenges with new routines*, *Difficult nursing decision*, and *Need of reflection and support*, highlight the changes in working conditions experienced by the nurses during the first wave of COVID-19 in Sweden, see Table 2.

**Table 2.** Themes and Sub-Categories.

Theme	*Sub-category*
Challenges with new routines	*Adaptation to new guidelines*
	*Challenges in using rigorous protective equipment*
	*Lack of training*
Difficult nursing decisions	*Challenges with priorities when patients deteriorated*
	*Nursing care*
Need of reflection and support	*Reflections and ethical thoughts*
	*The need of support*

### Challenges With New Routines

The healthcare organization rapidly changed and focused on taking care of patients who could not manage their symptoms at home. Support for those who could manage at home with professional medical advice was available through telehealth. At the primary care center, patients with symptoms of respiratory infection were assessed and treated in an outdoor tent in order to prevent the spread of infection. Home health nurses continued providing care according to previous routines, but patients with suspected COVID-19 symptoms were usually sent to hospital.

It went very fast from one day to the next. Then we reorganized our routines and all infected patients with upper respiratory tract infection for example cough, fever and other infection symptoms were assessed out in a triage tent. Inside the health center we have no patients with symptoms related to COVID 19. (p. 6)

At the hospital COVID-wards routines quickly changed, resulting in the need to develop and implement brief educational courses while managing heavier workloads, and ever-increasing stress levels. The new routines sought to develop efficient processes including, that is, creating an observational ward, increasing the number of hospital beds, moving patients to other somatic- or geriatric wards, transferring patients to infection wards, or discharging them.

The interviews revealed a sense of positive collaboration within the medical team. This teamwork became the glue that kept not only different departments and clinics together, but also various hospitals and primary care centers. When the COVID ward needed to transfer patients to other wards due to new incoming patients, the transfer process was performed more effectively. To adapt to the new situation of an increasing need for hospital care for patients with COVID-19, some clinics were closed in order to provide the new COVID-wards with the nursing staff. Nurses were transferred to the COVID departments. Although the nursing staff collaborated and helped each other, they also felt abandoned and left to solve complex situations, as demonstrated below:

We were thrown into it; we were told that we would get staff who would take care of monitoring patients, that we would get backup from staff at higher levels of care, but they never came. (p. 4)

#### Adaptation to New Guidelines

The results showed that due to the high number of patients and increased workload; nurses felt it was difficult for them to keep up with new guidelines and information from their employer, the WHO, and the Swedish public health agency. All nurses needed to quickly synthesize ongoing updates and update their routines. This was experienced as very time consuming.

There was no 100% reliable information, and we were directed to check the WHO and the Public Health Agency websites and constantly follow the internet for new information in order to stay updated. (p. 4)

Guidelines regarding which person protective equipment (PPE) to use and the availability of protective materials changed daily, leading to an uncertainty about how and which PPE to use in different patient situations. Furthermore, there were many unknowns about the function and adequacy of the PPE available. Were they sufficient to prevent transmission of the virus? The guidelines changed depending on the shifting information regarding how COVID-19 was transmitted, but also due to the lack of availability of protective material, as stated below:

I am responsible for infection control at my work, so I have regular contact with Infection Control Stockholm, regarding what guidelines to apply, what kind of PPE we need, how we can get PPE that’s needed. There were quite a lot of shortages at the beginning, and we needed to know how to use the protective clothing when there is a shortage, and how to protect the staff. (p. 3)

Infection control and hygiene routines during the pandemic were basically the same as prior to the pandemic. However, the application of rigorous hygiene routines was now in focus, as everyone became more aware of how the infection could spread on surfaces and between people. Nurses previously did not adhere to hygiene routines as carefully and rigidly as they did now.

I think the entire COVID-19 pandemic probably has occupied your thoughts, and therefore you have become more aware that material can spread infection, and this has led to an increased competence about it. (p. 2)

When patients were discharged from hospital to nursing care in the home it was unclear how long patients remained contagious. Wearing PPE became a new routine until the patients were asymptomatic for at least one week. Another new routine was wearing a face shield in every patient situation in order to protect elderly patients who were isolated at home and particularly vulnerable to infection. Increasing awareness led to careful and more frequent disinfection of surfaces and hands.

#### Challenges in Using Rigorous Protective Equipment

Guidelines regarding protective equipment were followed in accordance with the Swedish Communicable Diseases Control Agency's recommendations and the Swedish Public Health Agency. The materials that were used in close contact with patients, with suspected or confirmed COVID-19, were surgical masks or medical face masks with droplet filtration, face shields, short-sleeved or long-sleeved plastic aprons or gowns, and gloves. Furthermore, nurses had to continuously conduct their own risk assessments for transmission with each patient contact.

These guidelines, which now are the most recent, are as I have understood … is that if you should say that the patient has a CPAP treatment, or NIV, or nasal high-flow mask, or anything really where it is suspected that aerosols may be formed, … you have to use these FFP2 protections as it is now, but we used FFP3 in the beginning. Then they revised it from the region to use FFP2, visor, long-sleeved apron, gloves, so to really protect the body from splashes. (p. 7)

There were changes regarding putting on and removing PPE. It took time to learn how to properly use PPE without accidentally contaminating oneself. Nurses needed to prioritize their work and plan all care for the patient in advance before putting on PPE in order to save time and materials. They also had to make decisions about when to use the equipment.

We have never worked in such protective clothing where you have to meet each patient with a visor and face mask, and where you need to change protective equipment between each patient. (p. 3)

In home healthcare, nurses felt unaccustomed to caring for patients fully dressed in protective equipment. The homecare nurses needed to plan which protective equipment they needed to bring along, how much, and where, as well as how to change the protective equipment before entering a patient's home

There are guidelines on how you should put on and off the protective equipment, and you should take off one thing first and then the other and then use the hand sanitizer. Thus, you’re standing there with your coat behind your knees and the bag on a dirty floor … (p. 5)

#### Lack of Training

The rapid development of new guidelines and work routines allowed for little to no time for proper training. The nurses had to rely on web-based training, lectures, and tutorial videos found on YouTube. The training included hygiene routines, protective equipment, the spread of infection, and the disease process. Due to the high workload, it was not possible to train on relevant topics before the implementation of new routines and guidelines.

We have not received any real training; I can’t say that. It's more of a demonstration. (p. 2)

If you introduce new devices in a proper manner, a sales representative usually comes to the unit and introduces the device, and during a period you can test, feel, and press on the device so you can ask questions if there is anything that is unclear. We have never had that possibility, because we only received a pamphlet describing how to start up the device. (p. 1)

The excerpts above show that learning by doing prevailed in this environment of managing patients with a new virus. For example, the nurses in the COVID-department monitored oxygen saturation through a central monitor without instructions. Furthermore, respiratory support machines were delivered to the ward with simply an instruction sheet and no opportunities for hands-on training. Due to the high workload, nurses had to learn to use this equipment simultaneously while using them on patients. Due to the rapid reorganization and the emergent nature of the pandemic, nurses have been forced to perform work routines and tasks without training.

### Difficult Nursing Decisions

New ways to work with patients were established. Assessments by telephone, and asking questions about potential COVID-19 symptoms became a part of ordinary patient care. Since the most common symptoms of COVID-19 were fever, chest- or muscle pain, fatigue, or loss of sense of taste and smell, the initial triage was conducted by telephone with the goal of correctly guiding to the appropriate level of care. Patients who were directed to self-management at home received a telephone follow-up call by an RN to assess whether the patient had become worse and needed hospital care. Priorities were also made by using the L-ABCDE assessment tool, where *L* stands for life-threatening situation, A-airway, B-breathing, C-circulation, D-disability, and E-exposure, which assisted the nurses in their decision-making.

We created a template for how we should assess patients by telephone as many people call the primary care center for medical advice. It guides us on how we should assess the patients by telephone; if they needed to come to us for an assessment, who we should give self-care symptom management and advise to stay at home, or who we should send directly to hospital. (p. 6)

However, certain patients were difficult to safely assess over the telephone because it could lead to providing incorrect medical advice. Physical assessments in these cases took place at the primary care center or in the home, using the instrument L-ABCDE. The assessments were made by nurses or physicians and consisted of various parameters. This assessment guided decisions about whether the patient could be managed at home or needed hospital care.

The most difficult thing is to assess a patient before you have seen the patient, if for example, the homecare service has called, I’ll have to prioritize and assess the patient based on their history or report. (p. 2)

COVID-19 infection progressed differently in many patients, and it became clear that deterioration could occur several days after symptom onsets. When patients worsened, they developed severe respiratory problems, and the majority needed hospital care. Patients with respiratory insufficiency also displayed confusion and anxiety. Vital signs were monitored regularly and frequently, including venous sampling and blood gas to assess the severity of the patient's respiratory failure. Monitoring of oxygen saturation was also used to assess unstable patients.

A respiratory failure is usually what makes them worse—that they can’t maintain an optimal oxygen level. (p. 7)

Furthermore, the results showed the importance of triage at the primary care centers to assess if a patient needed hospital care or could manage at home. This triage contributed to a decreased burden for the emergency departments, which would not have been the case if the primary care centers had not developed a triage for suspected COVID-19 patients. There were challenges in accessing updates and new information while the patient intake was constantly increasing. Nurses worried about the protective equipment; if it would provide adequate protection enough or if the department would run out of protective equipment. Despite the challenges in gathering and disseminating information and the stressful work environment, several nurses emphasized the importance of cooperation. The triage was highlighted as contributing to more effective assessments and patient management.

#### Challenges With Priorities When Patients Deteriorated

At the onset of the COVID-19 pandemic, many patients deteriorated quickly, and this contributed to a shortage of hospital beds requiring higher levels of nursing and medical care, which was very challenging for the nurses. Patients were prioritized based on who should receive a higher level of care. Patients who deteriorated but were not prioritized to a higher level of care such as the intensive care unit remained in a COVID-ward. Consequently, these patients required more medical- and nursing care which was very time consuming and challenging.

Patients can be in a much worse condition than before and given the limited capacity at a higher level of care, leading to patients who previously shouldn’t have stayed with us with an oxygen saturation of 85%, for example. They should have been moved to intensive care units. (p. 4)

Many patients were prioritized to a lower level of care and classified as a palliative when there were not sufficient hospital beds. The homecare nurses describe a mental stress when they had to prepare for the possibility of managing palliative COVID-19 patients at home, causing ethical doubts to arise. The plan was to assess and prioritize patients at home based on a fragility scale: whether the patient was seriously ill and whether the patient would survive in hospital care, and whether the patient would remain at home. This plan was extremely challenging from an ethical point of view, as in Sweden, everyone usually gets the care that is needed. This plan was however not implemented and all sick patients requiring hospital care were instead cared for in hospital.

We, in the home care, found out that we should prepare to care for palliative COVID infected patients at home, if they might not get a place in a hospital. During the first weeks, we were completely sure that we would get dying patients’ home, who did not get a place in the hospital, and that we would not be able to provide the care they actually needed with oxygen and everything. This was mentally very heavy indeed. (p. 5)

The results showed that nurses experienced both physical and mental stress due to difficult prioritizations of care for their patients. All patients were affected, both those who had COVID-19 and those who had other diseases, since the normal level of nursing care was reduced. In hospitals and in homecare, prioritizing their tasks and patients’ needs has been key to keeping the healthcare system up and running during the initial stages of the pandemic. The medical center specialist clinics closed and patients with other diseases were forced to wait for their care.

#### Nursing Care

The most prominent nursing care were respiratory-, nutrition-, or psychosocial related. Nursing interventions were, that is, respiratory support, frequent changing of position to promote better oxygenation, calming conversations, and being present with the patient. The patients suffered from cough, infections, and poor oxygen saturation, which led to anxiety. Nurses needed to administer oxygen and different medication, that is, inhalations, antibiotics, or respiratory support, NIV or NHF and at the same time provide a calming presence.

They often need oxygen. They suffer a lot from respiratory distress, and they might have another infection when you hear wheezing sounds from the lungs while inserting a needle. They need a lot of chatting and are very worried. (p. 3)

Besides the anxiety caused by respiratory insufficiency, many patients worried about what they had heard about COVID-19. The increasing number of deaths raised many questions. The nurses had to mitigate the patients’ psychosocial anxiety and remain calm while being able to provide relevant information. One challenge was to remain calm, while using a rather loud voice behind the face mask and show empathy without facial expressions due to the protective equipment. Nurses developed ways to express empathy and relay calm using body language, for example, by sitting down by the patient and putting a hand on the patient's hand.

Of course, this was on the front page of all newspapers and on the news and everything for several months … so everyone has … all patients knew about this and had heard about what a terrible disease this is and everything … and the worry and anxiety the arriving patient has …, you must take it seriously. (p. 6)

Although many patients recovered and could be discharged to home, the recovery period for many patients was long. Some needed temporary help from primary care centers for medications, that is, injections of low molecular heparin to prevent thromboses. The nurses at homecare experienced that many patients in post-hospitalization were fatigued and had become thin.

### Need of Reflection and Support

The beginning of the pandemic led to new guidelines and routines, as well as an increased workload. The need of ethical reflection was evident. There was shortage of medical equipment that put the nurses in difficult ethical situations. All nurses interviewed expressed that they received support from their work teams and colleagues if problems occurred or to simply reflect on new situations. This theme refers to two subthemes: reflections and ethical thoughts and the need of support.

#### Reflections and Ethical Thoughts

Caring for patients with COVID-19 generated many situations for nurses where they had to reflect upon the difficulty and ethical challenges of their work, that is,—*Did I do the right things?—What went wrong?—How did I manage*? Many feelings and thoughts were shared with colleagues, and they provided essential support to each other. A sense of uncertainty regarding the protective equipment was a recurring concern: when PPE ran out and nurses found innovative ways to protect themselves and their patients by making their own PPE other questions arose: would they be adequately protected? Could they transmit the virus to other patients? To their colleagues and families? A constant onslaught of new information regarding how the virus could spread also increased concern, particularly around the asymptomatic infection, and transmission.

Since nurses were not regularly tested at the beginning of the pandemic due to a shortage of tests, they wondered if they were infected and if they would infect their patients*. These thoughts were ethically challenging as they were needed at work but might transmit the disease to others.* It also led to comparisons of symptoms between colleagues who had been ill. One nurse stated that if healthcare professionals had been tested earlier, the fear of being infected or infecting other patients would have diminished.

But now we, the staff, go around instead with an uncertainty about whether you have been infected or not and you almost sit and compare symptoms with each other. (p. 4)

The nurses reported being tired due to the rapid change of tasks and heavy workloads due to COVID-19 patients. When the nurses talked about their work, they described stressful working conditions. These radical changes in work made it feel like working in an entirely different workplace. Many patients were unstable and critically ill and not all nurses had previous experience of working with such patients.

It feels like you have a completely new job, and you get very tired, and you learn new routines, and now it has been a lot of extra work and it changes from day to day. (p. 3)

The nurses in the COVID-ward had to learn new tasks and use new medical equipment without training. This resulted in stress regarding patient safety issues, that is, managing new machines without any instruction. When nurses were afraid or had a problem in a certain situation, they turned to each other for support. In general, nurses felt that they could rely on their colleagues for support.

But I think it can contribute to stress when you should be able to use equipment that you don’t really know how to handle, and then the patient might get worse and die. Would it be my fault because I might have used the equipment as it wasn’t intended to, and therefore, it wouldn’t have had any effect. (p. 1)

Nurses expressed fear due to uncertainty and stress during the work shift. The high workload and continual need to learn new routines led to increasing fatigue.

#### Need of Support

The collegial support was crucial to maintaining resilience on an everyday basis. Daily meetings with the teams and colleagues offered opportunities to debrief as well as remain updated during the ongoing pandemic.

We have talked about guidelines, for example, recent ones, that you think are a bit odd, and you can ask your colleague how they perceive them. If they have come across a problem before, that I experience right now. Or how should I handle this new equipment that I’ve never used before. (p. 1)

Support was offered from the health care organization, that is, contact with a counselor or the hospital chaplain, but it was the nurses who must initiate contact with the right person and book a time when it was possible to leave the ward.

They have also told us that the counselors are there if you need to talk to someone and the hospital church has weekly discussion sessions for staff. (p. 4)

Nurses experienced support from their colleagues, families, and friends. They were eventually also offered access to professional help outside the hospital if necessary.

## Discussion

The purpose of this qualitative interview study was to describe nurses’ experiences in taking care of patients with suspected or confirmed COVID-19. This study was conducted at the very beginning of the pandemic 2020. It describes a specific period of time when there were new treatments and new guidelines for handling and in patients with COVID-19. Nurses had to manage patients without previous experience of the patient group. They had an enormous responsibility regarding hygiene routines, not spreading the infection, caring for the patients, etc. This interview study was performed in primary care settings and in hospital settings to capture both perspectives. The results show that the nurses had to work in a completely new situation, where guidelines constantly changed and they had to improvise protective equipment, prioritize patients by telephone instead of meeting and assessing them.

Furthermore, the results show that the experiences of nurses in primary care and hospital care did not differ, as both groups experienced rapidly changing work tasks, new routines, and ethical problems. They had to adjust quickly to new guidelines, new protective equipment, hygiene routines, etc. The collaboration with colleagues and team members became very important and highly regarded. They consulted colleagues when assessing patients’ conditions and it was important to prioritize patients for the right level of care. The rapid changes COVID-19 meant for the nursing care affected nurses and caused psychological distress. These results are confirmed by studies done in various countries (Barello et al., 2020; Crowe et al., 2021; Flateau et al. 2021; Fernandez et al., 2021; White et al., 2021).

### Organization and Responsibility

The pandemic put the entire health care system and all its functions through an enormous stress test. Every health care worker, at every level, became involved in the pandemic. Every routine was scrutinized. Everything that could be done faster was performed even more efficiently by removing other aspects of nursing care. The organizations’ routines changed rapidly from, that is, encountering patients in real life to assessing them by video online or by telephone. The results show how patients were instructed to self-manage their symptoms at home in a completely new way. As patients with mild symptoms were sent home with self-management instructions and information on when to return to care while those with severe symptoms were admitted to hospital. These routines were implemented, and research showed that early diagnosis, isolation, and symptom management could decrease the spread of the virus (Shahriarirad et al., 2020). However, patients with mild symptoms who worsened after a week came back for a new assessment and triage and those with severe symptoms were then referred to hospital. Sometimes nurses at homecare referred the patient directly to hospital, which also was the case in a study by White et al. (2021). This is something that can be used more in current health care routines. The results highlight the need of education to support quick changes to routines as well as ensuring that health care personnel are given information and are updated in order to support teamwork and decision making in the clinic. As nurses at the COVID-19 ward worked in accordance with new guidelines that rapidly and repeatedly changed over the initial course of the pandemic while also caring for patients in need of intensive care but due to the lack of beds in intensive care units, patients had to stay in intermediate care where sometimes enough NIV-ventilators were unavailable. It is an important finding that education and some training came with these changes, although the information given was scarce and the educational content shallow and brief due to the novelty of the virus and the emergent nature of the situation. It shows how evidence can be implemented in work routines in the clinic quite fast when there is an urgent need to develop new routines of treatment and care to secure patient survival. Some of these new routines and new evidence are important to evaluate further to enhance the quality of care. As the pandemic emerged people died and nurses witnessed their patients die, including patients who could have survived at a higher care level but died due to a lack of intensive care beds. Similar findings are presented in a study by Phua et al. (2020) which described the shortage of ventilator support, medication, intensive care wards, etc., as frustrating for the healthcare staff and forced them into ethical dilemmas. This finding highlights the importance of having routines in place to handle stressful situations including time for reflection and psychological support. This is not only related to pandemics, but stressful conditions and ethical dilemmas occur on most days as a nurse. It is a question of a sustainable working climate and being able to continue working for many years. Nurses and other health care staff will always be put in ethically difficult situations. If these challenges are not handled properly and space is not given to the staff to discuss, reflect and improve care, competent staff might eventually leave their workplaces due to burnout. The result of the present study showed a lack of ethical and practical guidelines for medical staff to follow in times of the clinical trials conducted during the pandemic, leaving the nurses in ethical distress and feeling completely exhausted. The results showed that nurses valued teamwork with colleagues highly and described the importance of trust and support from each other when they needed to talk and reflect. These findings are supported in a study by Haas et al. (2020), where nurses also emphasized the importance of a quiet place to rest.

### Reduce the Spread of Infection

Research has shown that COVID-19 is very contagious and can be transmitted indirectly as well (Arabi et al., 2020; Duncan & Lyall, 2020). Therefore, it was extremely important to use protection equipment and follow rigorous hygiene recommendations for the nurses to protect themselves and other patients. However, there was a shortage of protective equipment which complicated the work situation, leading to worries about becoming infected and infecting others. The lack of protective equipment was evident globally (Phua et al., 2020) and was a reality at the very beginning of the pandemic. The nurses in our study narrated that they had to reuse face masks and breathing masks due to shortages. Something that put them at a great risk of becoming infected as the risk for self-contamination is high when reusing protective equipment (Phua et al., 2020). This fact was very challenging for the nurses in our study as well and they were afraid of spreading the infection to others. The nurses in our study reported that they were unsure of how this level of protection equipment should be used and would have needed information and practice before using PPE in patient care. They had received scarce information or training on how to use it, which contributed to the feeling of anxiety and uncertainty among the nurses. However, this study was performed at the very beginning of the pandemic which should be considered.

### Strengths and Limitations

The study was performed at the beginning of the pandemic, and it was difficult to recruit participants, due to the extremely high influx of patients and workload. Healthcare staff were mandated to work extra hours to maintain patient safety which was stressful. Therefore, the number of participants in the study was limited. In addition, since the risk for transmission of COVID-19 was high, the interviews were performed online which could be a disadvantage. However, the interviewer was familiar with the online video program and could handle the interview situation well. The authors, therefore, suggest exploring this method further when interviews are used as data collection. The video interview also gives the researcher the possibility to watch the interview again, not only the sound. The validity of the study was assured as the participants were given the option to read through the transcribed interview before analysis, in accordance with Polit and Beck (2017). In order to further ensure the validity and the reliability of the study a semi-structured interview guide was used, and a pilot interview was performed to test the interview guide. We also used the COREQ checklist when planning and reporting the study (Tong et al., 2007). The transferability of the study was confirmed as we chose to include participants from both primary care and inpatient care. However, we did not have participants from intensive care which would have been beneficial when describing the experiences of nurses at different care levels.

### Implications for Practice

The result shows the importance of updated clinical guidelines and the implementation of those guidelines to reduce infection spread, avoid uncertainty among staff and maintain patient safety. Furthermore, the result highlights the importance of functional teamwork and organization to maintain a trustful and safe working environment. Easy access to support and giving time for reflection are also important to provide.

## Conclusion

Nurses had to quickly adjust to entirely new routines and develop ways of providing safe nursing care in a continuously changing medical environment. Many situations led nurses to wonder if they had made the right or wrong decision. Support from colleagues was crucial to be able to continue working during the initial stage of the pandemic. The nurses experienced that many patients deteriorated, leading to a difficult and exhaustive medical and caring situation. They also experienced increasing responsibility since they had to implement new protective equipment and work according to new recommendations, making them feel uncertain about patient safety.
